# Effects of age and tissue of *Juniperus sabina* L. on its phytochemical characteristics, anti-cholinesterase, antidiabetes, and anti-drug resistant bacteria activities

**DOI:** 10.3389/fpls.2023.1174922

**Published:** 2023-09-04

**Authors:** Shengnan Xu, Qian Chen, Na Luo, Jinyan Yang, Dengwu Li

**Affiliations:** ^1^ College of Forestry, Northwest A & F University, Yangling, Shaanxi, China; ^2^ Shaanxi Key Laboratory of Economic Plant Resources Development and Utilization, College of Forestry, Northwest Agriculture and Forestry University, Yangling, Shaanxi, China

**Keywords:** *Juniperus sabina* L., phenols, lignans, neuroprotection and antidiabetic activities, anti-bacteria activities

## Abstract

*Juniperus sabina* L. is used in the traditional Chinese medicine (TCM) system to prevent or treat various diseases. However, only the leaves and branches are used as medicinal parts. The aim of this study was to compare the chemical characteristics of different tissues (leaves, branches, stems, and roots) of *J. sabina* at different ages by HPLC-MS and to evaluate the biological activity (enzyme inhibition, anti-drug-resistant bacteria). Total phenol (TPC) and total lignan (TLC) contents in *J. sabina* were determined by Folin-Ciocalteu method and UV spectrophotometry, respectively. High levels of total phenols (87.16 mg GAE/g dry weight) and total lignans (491.24 mg PPT/g dry weight) were detected in fifteen annual *J. sabina* roots and current year leaves, respectively. Eleven compounds, of which six were phenolic compounds and five were lignans, were identified and quantified by HPLC/HPLC-MS. Statistical analysis showed that the distribution and content of the detected compounds showed considerable variation among ages and tissues, and that the current year leaves of fifteen annual *J. sabina* could be used as a potential application site for the source of podophyllotoxin. Acetylcholinesterase (AChE) inhibitory activity was found to be the highest on the extracts of fifteen annual *J. sabina* current year leaves (47.37 μg/mL), while the highest inhibition towards butyrylcholinesterase (BChE) was observed for the extracts of seven annual *J. sabina* previous year leaves (136.3 μg/mL). And the second annual *J. sabina* current year stem’s extracts showed the best antidiabetic activity (anti-α-glucosidase, 62.59 μg/mL). In addition, the extracts of fifteen annual *J. sabina* roots (47.37 μg/mL) showed the highest anti-MRSA activity (31.25 μg/mL). Redundancy analysis (RDA) was conducted to clarify the factors affecting the biological activity of *J. sabina*, and its results showed that epicatechin and matairesinol showed positive promotion. This study provides a new perspective for understanding the chemical differences and comprehensive utilization of different tissues of *J. sabina*.

## 1 Introduction

*Juniperus* L, represented by 70 species in the world and 23 species in China, belongs to the Cupressaceae family (Esteban et al., 2023; Kavaz and Faraj, 2023; Lim et al., 2023). The wood, bark and leaves of the juniper genus are rich sources of natural products that can be used as fragrances in alcoholic beverages and cosmetics, with high commercial application value (Rangel et al., 2018). Previous phytochemical studies of *Juniperus* species showed a rich chemical content, including diterpenes, triterpenes, phenolic acids, flavonoids and lignans (Tavares and Seca, 2018; Lim et al., 2023). The relevant *in vitro* and *in vivo* studies indicated that many *Juniperus* species possess anti-inflammatory, antioxidant, antiviral, antibacterial, hypotensive, anticancer, antidiabetic, and neuroprotective properties (Al-Snafi, 2018a; Al-Snafi, 2018b; Gonçalves et al., 2022; Rehman et al., 2022; Spengler et al., 2022; Kavaz and Faraj, 2023).

*Juniperus sabina* L. is an evergreen coniferous shrub in the *Juniperus* genus, widely cultivated as a horticultural plant and used for decoration in China (Wang et al., 2014). *J. sabina* is rich in volatile oils, phenols and lignans (Xu et al., 2022). In particular, *J. sabina* is rich in podophyllotoxin, a synthetic precursor of the first-line anticancer drug etoposide (Ivanova et al., 2023). However, current studies on the genus Juniperus have mainly focused on *Juniperus communis* L. and *Juniperus oxycedrus* L. (Majewska et al., 2017; Spengler et al., 2022). Although data on the chemical composition of phenols and lignans from *Juniperus* species cones and leaves have been published, most studies focus on the isolation and characterization of single compounds (Xu et al., 2022). Furthermore, the content of phenols and lignans in plants and their biological activity depend on age, tissues, and ontogeny stage (Dossou et al., 2021; Butnaru et al., 2022). The phytochemical and molecular characteristics of perennials, especially medicinal plants, vary significantly with age and this must be taken into account (Bielecka et al., 2019). To our knowledge, there is currently no literature data on the composition of compounds or their biological activities or medicinal properties in different tissues of different ages *J. sabina*. Considering the importance of multiple enzymes, such as cholinesterase and α-glucosidase, in the development of many chronic diseases, it is worthwhile to study the enzyme-inhibitory activity of *J. sabina.* Moreover, almost all *Juniperus* species show antibacterial activity, but the studies are only for common strains, and the research on the inhibition of drug-resistant bacteria is still lacking.

Based on this, this study aims to analyze chemical composition of different tissues (leaves, branches, stems, roots) of *J. sabina* at different ages and evaluated their biological activities (enzyme inhibition, antibacterial activity) to address the key and difficult issues in *J. sabina* applications. These results can promote the comprehensive utilization of *J. sabina* plant resources.

## 2 Material and methods

### 2.1 Plant materials

**Figure 1:**
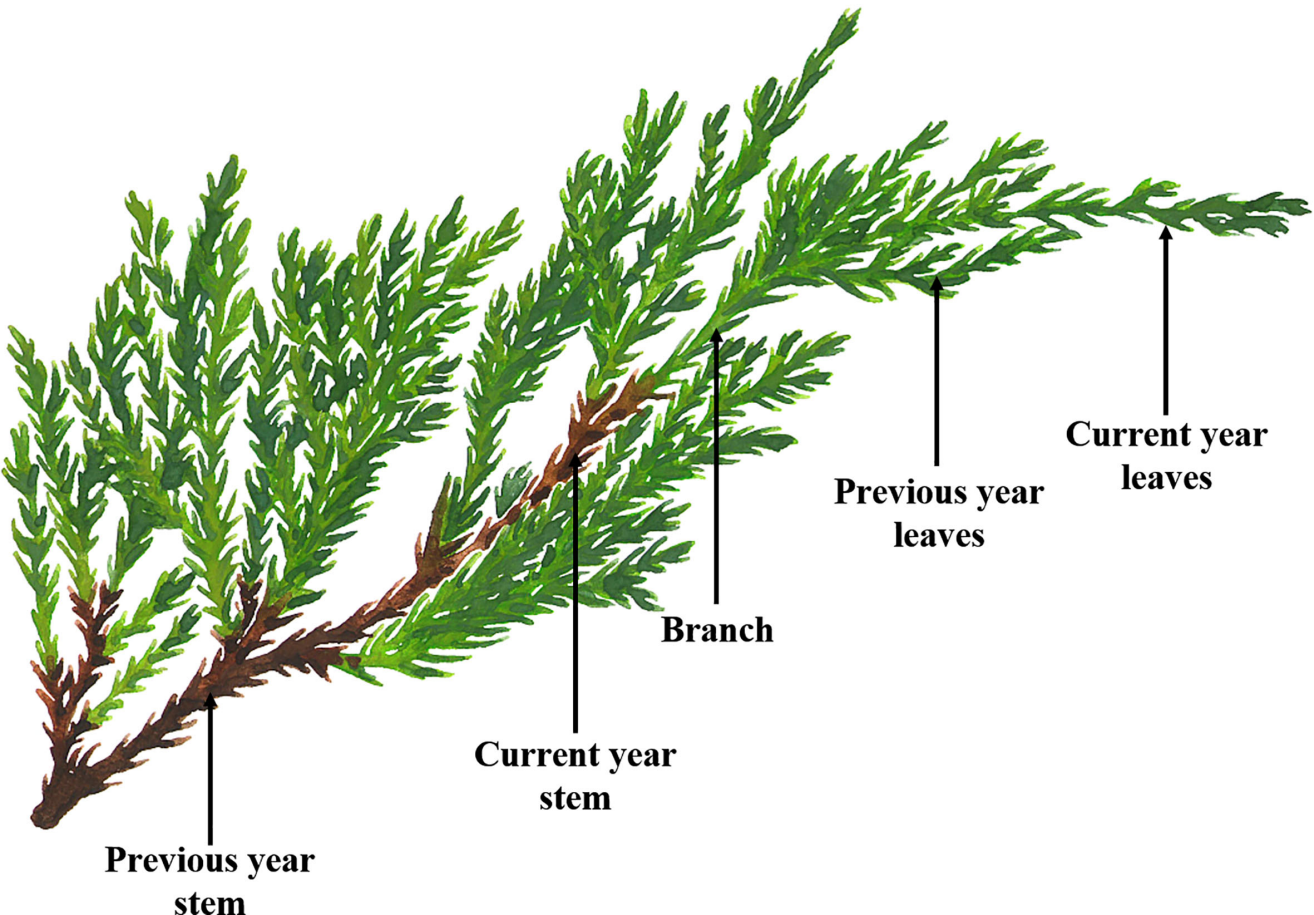
Schematic diagram of *J. sabina* samples.

In this study, three different tree ages (biennial, seven annual and fifteen annual) of *J. sabina* were selected as test materials. The test samples were identified by Prof. Kunliang Dang of the College of Forestry, Northwest A&F University, and kept in the herbarium of the College of Forestry, Northwest A&F University. Five plants with good growth and healthy trees were selected as target trees before sampling. Samples were collected in July 2020 from two experimental sites (Northwest A&F University School of Forestry Nursery and Northwest A&F University Expo Park). According to the physiological and morphological characteristics of the collected samples (Cheng et al., 2005), the current year leaves, previous year leaves, branches, current year stem, previous year stem and roots (**Figure 1**) were separated. The samples were prepared according to the method of “freeze-drying to constant weight, crushing and sieving (Pharmacopoeia standard sieve No. 4)”, and stored at -20°C.

### 2.2 Extraction procedure

The samples were extracted according to the method of Xu et al. (2022). Samples of different tissues (5 g each) of *J. sabina* were weighed into triangular flasks, and 200 mL of a 90% methanol-water solution was added. The mixture was treated in an ultrasonic bath (HH-6, Shanghai High Precision Instrument Co., Ltd., Shanghai, China) for 7 min, followed by homogenization-assisted extraction at 55°C for 90 min using a constant temperature magnetic stirrer (XMTD203, Jiangsu Keyi Instrument Co., Ltd., Nanjing, China). The extracts were filtered and evaporated under vacuum at 45°C until dryness. The crude extracts obtained were kept at 4°C prior to the activity and phytochemical experiments. The yields (%, w/w) of the crude extracts were given in **SM-Table 1** (**Supplementary Material**). Extracts of plant material were performed in triplicate.

### 2.3 Determination of total phenols and lignans

The phytochemical analysis of extracts was performed according to the method described by Gómez-Martínez et al. (2020) with some modifications. The total phenol content (TPC) of the extracts was determined according to the Folin-Ciocalteau method. After pipetting 200 μL of extracts of *J. sabina* (1 mg/mL) into a test tube, 2 mL of Folin-Ciocalteu reagent was added, followed by a 7% Na_2_CO_3_ solution. The mixture was shaken well and left to react for 6 min before reading the absorbance at 760 nm on an ultraviolet spectrophotometer (UV-1800, Shimadzu Manufacturing Co., Kyoto, Japan). A standard curve (**SM-Figure 1A**) was drawn with gallic acid dilutions, and the results of TPC were expressed as mg gallic acid equivalents (GAE) per g of sample dry weight (mg GAE/g DW). The results were expressed as mg of gallic acid equivalents (mg GAE) per g of dry weight (DW).

The total lignan content (TLC) of the extracts was calculated by UV spectrophotometry. Briefly, serial dilutions of podophyllotoxin (PPT) were used to prepare standard calibration curves. The extracts (1.25 mg/mL) and podophyllotoxin dilutions were measured for absorbance at 290 nm with a UV-Vis spectrophotometer (UV mini-1240, Shimadzu Manufacturing Co., Kyoto, Japan). A standard curve (**SM-Figure 1B**) was drawn with podophyllotoxin dilutions, and the results of TLC were expressed as mg podophyllotoxin equivalents per g of sample dry weight (mg PPT/g DW).

### 2.4 Identification of phenols and lignans by HPLC/HPLC-MS analysis

#### 2.4.1 HPLC-MS analysis

The samples (leaves, branches, stems, and roots) of *J. sabina* were used for metabolomic analysis, with three biological replicates per group. The HPLC separation was carried out using an EXIONLC System (SCIEX, USA), and the column was a Waters Acquity UPLC HSS T3 (2.1 mm x 100 mm, 1.8 μm, Waters, USA). The mobile phases were 0.1% formic acid-water solution (A) and acetonitrile (B) with the following gradients: 0-0.5 min, 2% B; 0.5-10 min, 2%-50% B; 10-11 min, 50%-95% B; 11-13 min, 95% A; 13-13.1 min, 95%-2% B; 13.1-15 min, 2% B. The column temperature was set at 40 °C and the flow rate was 0.4 mL/min. The auto-sampler temperature was set at 4 °C and the injection volume was 2 μL.

A SCIEX 6500 QTRAP+ triple quadrupole mass spectrometer equipped with an IonDrive Turbo V ESI ion source was used for the mass spectrometry analysis in multiple reaction monitoring (MRM) modes. The ion source parameters were as follows: IonSpray Voltage: +5500/-4500 V, Curtain Gas: 35 psi, Temperature: 400 °C, Ion Source Gas 1:60 psi, Ion Source Gas 2: 60 psi, DP: ± 100 V. Data acquisition is performed in full scan mode in the range of m/z 50-1000. Analyst Work Station Software (Version 1.6.3, SCIEX, USA) was used for all mass spectrometry data acquisition and quantitative analysis of target compounds. The MS conventer software was used to convert the raw mass spectra into TXT format. Then the R program package was used for peak extraction and annotation in combination with a self-created database. MRM transitions and MS conditions for the identification of compounds were shown in **SM-Table 2**.

#### 2.4.2 HPLC quantitative analysis

**Figure 2:**
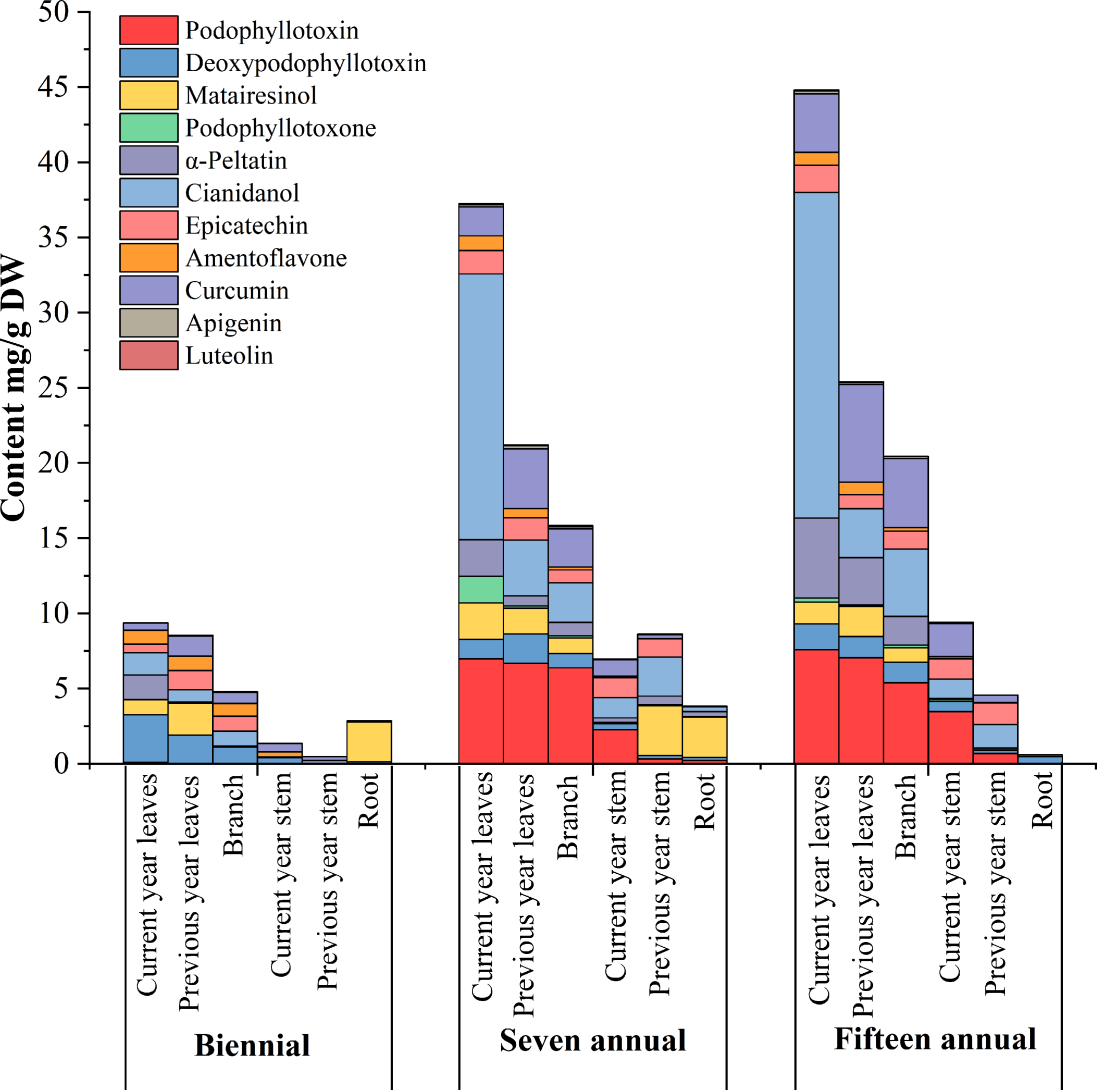
Accumulation analysis of compounds in different tissues extracts of different ages *J. sabina*.

The determination was performed by high-performance liquid chromatography (HPLC, Agilent 1260, Agilent Technologies Inc., Santa Clara, CA, USA) using a C18 (250 mm×4.6 mm, 5 μm; Agilent Technologies Inc., Santa Clara, CA, USA) column. The mobile phases were methanol (A) and 2% formic acid-water solution (B), and the gradient was as follows: 0-10 min, 10%-30% A; 10-20 min, 30%-55% A; 20-35 min, 55%-80% A; 35-40 min, 80%-84% A; 40-45 min, 84%-100% A; 45-50 min, 100% A; 50-56 min, 100% A. The column temperature was 35°C, the flow rate was 0.8 mL/min, the injection volume was 20 μL, and the UV detection wavelength was set to 290 nm. The contents of 11 compounds identified in this study were calculated by the external standard method, that is, by referring to the peak area of standard compounds. The standards for the 11 compounds in this study were purchased from Yuanye Bio-Technology Co., Ltd. (Shanghai, China). Standard solutions were prepared in methanol with an initial concentration of 1 mg/mL, and a standard curve was drawn after serial dilution (**SM-Table 3**, **SM-Figure 2**).

### 2.5 Enzyme activity inhibition test

#### 2.5.1 Cholinesterase inhibitory activity assays

A modified Ellman’s method was used to assess the AChE and BChE enzyme inhibitory activities of *J. sabina* extracts (Ellman et al., 1961). The extracts were dissolved as gradient dilutions using 2% DMSO water for activity determination. The extract gradient dilutions (1000, 500, 250, 125, 62.5, and 31.25 µg/mL) 20 μL were incubated with AChE (0.28 U/mL, 20 μL) and BChE (0.28 U/mL, 20 μL) in phosphate buffer (PBS, pH 7.8, 140 µL) for 15 min in 25°C. Afterwards, acetylthiocholine iodide and butyrylthiocholine chloride were added as the substrates (10 µL) for AChE and BChE, respectively. Simultaneously, the coloring reagent 5,5-dithio-bis (2-nitrobenzoic) acid (DTNB, 10 µL) was added and incubated at 37°C for 30 minutes. The absorbance values of the sample wells were measured at 412 nm. PBS buffer was used as a blank, and galanthamine was used as the reference drug. Analyses were performed in triplicate. The cholinesterase inhibitory was calculated according to the following equation:

Cholinesterase inhibitory (%) = (Blank group -Experimental group)/Blank group *100%

AChE (*electric eel*), BChE (*horse serum*), and galanthamine were all purchased from Sigma-Aldrich (Sigma, St. Louis, MO, USA).

#### 2.5.2 α-Glucosidase inhibitory activity assay

The α-glucosidase (α-Glu) inhibition assay was performed according to the method of Zaharudin et al. (2019) with a slight modification. The extract gradient dilutions (1000, 500, 250, 125, 62.5, and 31.25 µg/mL) 10 µL, α-glucosidase (2.0 U/mL, 10 µL) and PBS (pH 6.8, 80 µL) were added to a 96-well microtiter plate. The mixture was incubated for 10 min at 37°C. The *p*-nitrophenyl-α-*d*-glucopyranoside (20 µL) was mixed into the solution and incubated at 37°C for 20 min to initiate the reaction. The reaction was aborted by the addition of sodium carbonate solution (100 μL). Acarbose was used as the reference drug, and 2% DMSO water was used as the solvent control. Enzyme inhibitory was quantified by measuring the release of *p*-nitrophenol at 405 nm.

All experiments were performed in triplicate. α-Glucosidase inhibitory effect was calculated by the following equation:

α-Glucosidase inhibitory (%) = $\frac{\left({\text{A}}_{\text{K}}-{\text{A}}_{\text{K}0}\right)-\left({\text{A}}_{\text{S}}-{\text{A}}_{\text{S}0}\right)}{{\text{A}}_{\text{K}}-{\text{A}}_{\text{K}0}}\times 100$ Among them, A_S_ = sample absorbance value, A_K_ = control (2% DMSO water) absorbance value, and the background absorbance value of the reaction system was subtracted without enzyme in A_S0_ and A_K0_.

### 2.6 Antibacterial activity determination

Bacterial strains of methicillin-resistant *Staphylococcus aureus* (MRSA, ATCC4300) and vancomycin-resistant *Escherichia coli* (VREC) were provided by the Key Laboratory of Synthetic and Natural Functional Molecule Chemistry of Ministry of Education, Chemical Biology Innovation Laboratory, College of Chemistry and Materials Science, Northwest University (Chen and Yang, 2019). The extracts of *J. sabina* were dissolved in a 2% DMSO-water solution to prepare the samples to be tested at the corresponding concentrations and passed through 0.22 μm Millipore Express (Bio-protocol LLC., China) for later use. Antimicrobial resistance tests were performed according to CLSI microbiological test standards (CLSI, 2010). Specific experimental methods were referred to the adapted test method of Falcão et al. (2018). Individual colonies of clinical isolates MRSA and VREC on LB agar plates were transferred to 5 mL Mueller-Hinton (MH) liquid medium and grown overnight at 37°C. Bacterial cells were collected by centrifugation (4000 rpm, 10 min). After discarding the supernatant, the pelleted cells were resuspended in MH medium and diluted to an OD600 of 0.5. The sample solution (20 µL) and bacterial suspension (10^5^ CFU/mL, 180 µL) were mixed and incubated at 37°C for 18 h. Vancomycin hydrochloride and colistin sulfate were used as positive controls for MRSA and VREC, respectively. The microdilution minimum inhibitory concentration (MIC) was interpreted as the lowest drug concentration that completely inhibited the visible growth of bacteria (OD600< 0.1) after incubating the plates for at least 16 h at 37°C. Each inhibitor was tested in triplicate.

### 2.7 Statistical analysis

The assays described were performed in triplicate for all experiments and the results are expressed as mean ± standard deviation (SD). All experiments were repeated three times. Statistical analysis was made by one-way analysis of variance (ANOVA) in SPSS 18.0 software. Redundancy analysis (RDA) was carried out in R (version 4.1.2) using vegan package.

## 3 Results and discussion

### 3.1 Variation of TPC and TLC in *J. sabina* during ages

This study evaluated the content of total phenols and lignans in different tissue extracts from different ages of *J. sabina*. **Table 1** showed that among the tissues, the roots were richer in phenolic substances. The highest TPC (487.16 mg GAE/g DW) was found in root extracts from fifteen annual *J. sabina*. The total phenol content in leaves was also considerable, and it showed an increasing trend with the increase in tree age. The determination results of total lignans showed that TLC in *J. sabina* leaves was the highest (306.09-491.24 mg PPT/g DW), and it showed an increasing trend with the increase in tree age (**Table 1**). The highest total lignan content was found in the current year’s leaf extracts of fifteen annual *J. sabina*. TPC and TLC showed considerable differences in the age and tissues of *J. sabina*. The roots and leaves are the dominant tissues at different ages for the distribution of total phenols and total lignans, respectively.

**Table 1:** Contents of total phenols and total lignans in *J. sabina* during different ages.

Age	Tissue	Total phenols content a mg GAE/g DW	Total lignan content b mg PPT/g DW
Biennial	Current year leaves (BCL)	138.49 ± 0.8**	306.09 ± 0.12**
	Previous year leaves (BPL)	239.02 ± 1.1**	361.59 ± 0.33**
	Branch (BB)	199.19 ± 0.3**	236.76 ± 0.22**
	Current year stem (BCS)	240.42 ± 0.53**	100.25 ± 0.12**
	Previous year stem (BPS)	215.68 ± 0.53**	92.9 ± 0.12**
	Root (BR)	441.47 ± 0.53**	286.92 ± 0.22**
Seven annual	Current year leaves (SCL)	295.58 ± 1.05**	405.12 ± 0.25**
	Previous year leaves (SPL)	342.95 ± 1.05	342.27 ± 0.43**
	Branch (SB)	187.86 ± 1.61**	153.95 ± 0.37**
	Current year stem (SCS)	173.47 ± 1.05**	95.57 ± 0.22**
	Previous year stem (SPS)	412.07 ± 0.61**	125.26 ± 0.33**
	Root (SR)	327.16 ± 1.05**	377.95 ± 0.78**
Fifteen annual	Current year leaves (FCL)	390.67 ± 1.61**	491.24 ± 0.57**
	Previous year leaves (FPL)	345.05 ± 1.05	373.84 ± 0.43**
	Branch (FB)	242.6 ± 1.61**	180.76 ± 0.22**
	Current year stem (FCS)	197.68 ± 1.05**	117.62 ± 0.22**
	Previous year stem (FPS)	198.74 ± 1.82**	57.23 ± 0.12**
	Root (FR)	487.16 ± 2.11**	132.32 ± 0.22**

a: data expressed in mg equivalent of gallic acid (GAE) to 1 g of sample dry weight; b: Data expressed in mg equivalent of podophyllotoxin (PPT) to 1 g of sample dry weight; ** P<0.01.

Previous studies have shown that the main active substances in *J. sabina* were found to be phenols and lignans, and phenols are important secondary metabolites for plant resistance to biotic and abiotic stresses (Sarkar and Shetty, 2014; Tavares and Seca, 2018). In this study, the TPC of *J. sabina* was higher than that reported by Gonçalves et al. (2022). In addition, the lignans and their derivatives in *J. sabina* leaves exhibited excellent insecticidal and acaricidal activities (Yang et al., 2017; Yang et al., 2021). The diversity and heterogeneity of total lignans enriched in *J. sabina* leaves and roots provided broad-spectrum protective defenses against a variety of pathogens and pests. The lignans (podophyllotoxin, deoxypodophyllotoxin, etc.) in *J. sabina* were also shown to have anti-tumor and antimicrobial activity (Liu et al., 2021).

### 3.2 Identification of phenols and podophyllotoxins by HPLC/HPLC-MS

**Figure 3:**
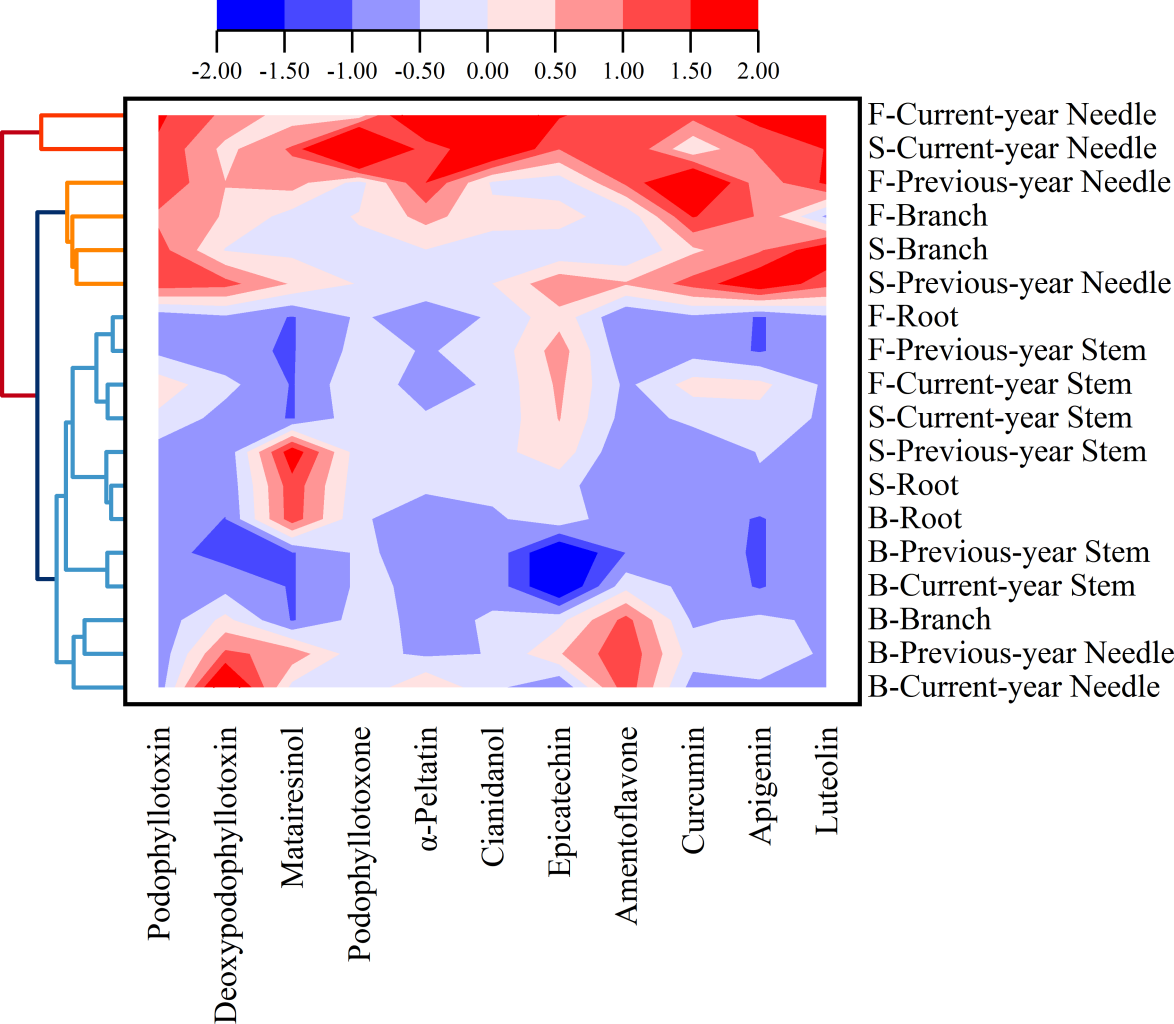
Cluster analysis of compounds in different tissues extracts of different ages *J. sabina*.

**Figure 4:**
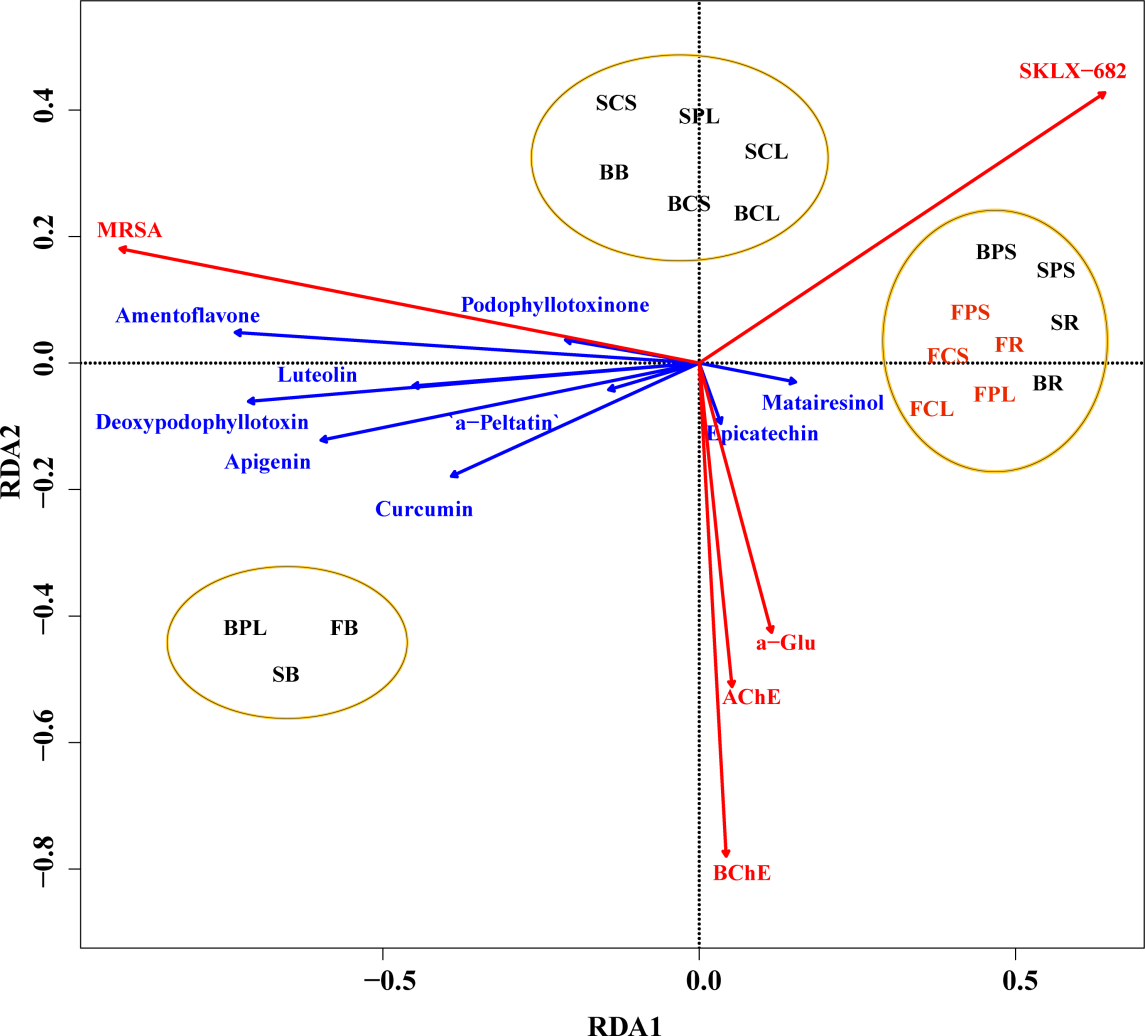
RDA analysis of *J. sabina* samples. Reaction variables (biological activity of the extract, marked by red arrow), explanatory variables (compound content, marked by blue arrow), sample classification cluster (marked by ellipse).

The HPLC characterization of *J. sabina* extracts allowed the identification of a total of 11 characteristic compounds, separated by their molecular mass and according to their retention time (**Table 2**) using the MS analyst Work Station Software (Version 1.6.3). Positive ionisation was used as the MS operating condition for the identification of the compounds. The present study showed that most phenols were more abundant in the tissues of adult *J. sabina* compared to juvenile *J. sabina*. The most common taxonomic substances included catechin (**SM-Figure 3A**), epicatechin and curcumin, followed by apigenin and amentoflavone, which were only found in tissues other than the roots of *J. sabina*, and luteolin, which was only found in the leaves of adult *J. sabina*. Compound 11 was identified as curcumin by comparison with the standards (**SM-Figure 3B**). In the (+) ESI-MS/MS analysis, the product ion m/z 177.1 was observed as the dominant peak (**SM-Figure 3B**; **SM-Figure. 4**), which is consistent with the characteristic ion peak of curcumin previously reported (Jia et al., 2017). This is the first time that curcumin has been found in juniper. The second most common group of compounds were the podophyllotoxins, which belong to the aryl naphthalene group of lignans, such as podophyllotoxin (**SM-Figure 3C**) (deoxypodophyllotoxin, matairesinol, α-Peltatin, and podophyllotoxinone). These compounds were identified in all ages of *J. sabina* leaves.

**Table 2:** Main compounds in *J. sabina* identified by HPLC/HPLC-MS.

CAS Number	RT (min)	Ion mode	Q (m/z)	q (m/z)	Compounds	Family	Biennial						Seven annual						Fifteen annual					
							BCL	BPL	BB	BCS	BPS	BR	SCL	SPL	SB	SCS	SPS	SR	FCL	FPL	FB	FCS	FPS	FR
154-23-4	4.50	+	290.9231	165.0012	Catechin1	Phenols	1.47 ± 0.11**	0.81 ± 0.11**	0.97 ± 0.31**	–	–	–	17.68 ± 0.14**	3.7 ± 0.54	2.63 ± 0.17**	1.35 ± 0.1	2.59 ± 0.21**	1.85 ± 0.32**	21.66 ± 0.08**	3.26 ± 0.30	4.48 ± 0.56**	1.3 ± 0.03	1.56 ± 0.01**	1.11 ± 0.00**
490-46-0	5.08	+	291.1002	139.0003	Epicatechin2	Phenols	0.58 ± 0.00**	1.29 ± 0.02**	0.99 ± 0.01**	–	–	1.01 ± 0.07	1.55 ± 0.13	1.49 ± 0.06**	0.86 ± 0.01	1.32 ± 0.18	1.22 ± 0.04**	0.92 ± 0.02	1.81 ± 0.28	0.93 ± 0.02**	1.19 ± 0.22	1.35 ± 0	1.43 ± 0.01**	1.22 ± 0.12
491-70-3	8.03	+	286.9004	240.9003	Luteolin5	Phenols	–	–	–	–	–	–	0.06 ± 0.00**	0.05 ± 0.00**	0.08 ± 0.00**	–	–	–	0.06 ± 0.00**	0.06 ± 0.00**	–	–	–	–
520-36-5	8.90	+	270.9002	163.0000	Apigenin8	Phenols	0.03 ± 0.02**	0.06 ± 0.01**	0.05 ± 0.01**	–	–	–	0.15 ± 0.02	0.21 ± 0.04*	0.14 ± 0.02	0.06 ± 0.01**	0.04 ± 0.01**	0.03 ± 0.02*	0.19 ± 0.03	0.12 ± 0.01*	0.14 ± 0.02**	0.08 ± 0	–	–
1617-53-4	9.78	+	539.0031	402.9001	Amentoflavone10	Phenols	0.92 ± 0.02**	0.95 ± 0.00**	0.86 ± 0.00**	0.33 ± 0.00**	–	–	0.98 ± 0.00**	0.61 ± 0.00**	0.19 ± 0.00**	0.11 ± 0.00**	–	–	0.85 ± 0.01**	0.83 ± 0.01**	0.24 ± 0.03**	0.15 ± 0.01**	0.04 ± 0.00	–
458-37-7	5.69	+	369.1008	177.1009	Curcumin11	Phenols	0.48 ± 0.01**	1.33 ± 0**	0.74 ± 0.01**	0.57 ± 0.00**	0.26 ± 0.00**	0.43 ± 0.01**	1.92 ± 0.00**	3.96 ± 0.00**	2.54 ± 0.10**	1.09 ± 0.00**	0.28 ± 0.00**	0.3 ± 0.00**	3.9 ± 0.05**	6.49 ± 0.04**	4.6 ± 0.07**	2.19 ± 0.01**	0.49 ± 0.00**	0.67 ± 0.00**
477-49-6	5.42	+	413.1001	395.1003	Podophyllotoxinone7	Lignans	0.01 ± 0.00**	0.04 ± 0.00**	0.05 ± 0**	–	–	–	1.79 ± 0.00**	0.16 ± 0.01**	0.14 ± 0.00**	0.1 ± 0.00	0.05 ± 0.01*	0.04 ± 0.01**	0.27 ± 0.00**	0.1 ± 0.00**	0.18 ± 0.00**	0.14 ± 0.00	0.04 ± 0.00*	–
518-28-5	9.66	+	417.2002	247.0601	Podophyllotoxin6	Lignans	0.09 ± 0.01**	–	–	–	–	–	6.99 ± 0**	6.67 ± 0.03**	6.38 ± 0.19**	2.27 ± 0.01**	0.32 ± 0.01**	0.21 ± 0.01**	7.58 ± 0.11**	7.05 ± 0.02**	5.39 ± 0.09**	3.47 ± 0.01**	0.68 ± 0.04**	–
580-72-3	9.12	–	357.1002	83.0002	Matairesinol3	Lignans	1.01 ± 0.00**	2.13 ± 0.00**	–	–	–	2.65 ± 0.06*	2.42 ± 0.05**	1.7 ± 0.04**	1.03 ± 0*	–	3.33 ± 0.21**	2.68 ± 0.35*	1.46 ± 0.02**	2.01 ± 0.07**	0.96 ± 0.39*	–	–	–
19186-35-7	10.06	+	399.1001	311.1001	Deoxypodophyllotoxin9	Lignans	3.16 ± 0.09**	1.9 ± 0.00**	1.1 ± 0.02**	0.4 ± 0**	–	0.13 ± 0.00**	1.28 ± 0.00**	1.96 ± 0.00**	0.95 ± 0.03**	0.39 ± 0.00**	0.22 ± 0.00**	0.21 ± 0.00**	1.72 ± 0.02**	1.4 ± 0.01**	1.36 ± 0.02**	0.69 ± 0.01**	0.21 ± 0**	0.48 ± 0.00**
568-53-6	12.59	+	401.1013	165.1012	α-Peltatin4	Lignans	1.64 ± 0.06**	0.04 ± 0.00**	0.04 ± 0.01**	0.05 ± 0.04**	0.21 ± 0.01**	–	2.42 ± 0.01**	0.68 ± 0.00**	0.89 ± 0.40**	0.29 ± 0.01**	0.58 ± 0.12**	0.33 ± 0.31**	5.31 ± 0.07**	3.14 ± 0.04**	1.91 ± 0.04**	0.03 ± 0.01**	0.12 ± 0.01**	–

a: RT- Retention times (min); all values are presented as the mean ± SD of triplicate determinations (n = 3); content is expressed as mg of raw material per g of dried weight; ** P<0.01, * 0.01<P<0.05.

The results of the quantitative analysis (**Table 2**) showed significant differences in the age and tissue distribution of individual phenol and podophyllotoxin compounds in *J. sabina*. The total distribution of 11 compounds in *J. sabina* of different ages was in the order: fifteen annual > seven annual > biennial (**Figure 2**), among which the more abundant substances were catechin, curcumin, podophyllotoxin and α-peltatin. The highest curcumin content (6.49 mg/g DW) was obtained in previous year’s leaves of fifteen annual *J. sabina*. The highest contents of the other three compounds were obtained from the current year leaves of fifteen annual *J. sabina*, which were catechin (21.66 mg/g DW), podophyllotoxin (7.58 mg/g DW) and α-peltatin (5.31 mg/g DW). The content of most of the tested compounds in the different tissues of *J. sabina* was in decreasing order: current year leaves > previous year leaves > branch > current year stem > previous year stem > root (**Figure 2**). The current data suggests that the content of phenols and podophyllotoxins may be highly dependent on the growth process of secondary metabolite transformation and biosynthesis in the organs of *J. sabina*, as in earlier studies on other plants (Bai et al., 2017; Feng et al., 2014; Kim et al., 2020).

The results of the clustering (**Figure 3**) of the 11 identified components in *J. sabina* showed that the extracts were divided into 3 groups: the current year leaves of seven and fifteen annual *J. sabina* were divided into Group I; the previous year leaves and branches of seven and fifteen annual *J. sabina* were divided into Group II; the remaining other tissues were divided into Group III. **Figure 3** showed that 11 compounds had higher content in Group I and Group II, mainly in the leaves and branches of adult *J. sabina*. The leaves and branches of *J. sabina* were the main medicinal tissues in traditional ethnopharmacological applications, which also showed consistency with our clustering results (Kavaz and Faraj, 2023). Significantly, the compounds detected in this study were low in young *J. sabina*, so age was probably a key factor in the quality of the medicinal tissues of *J. sabina* (Ghasemzadeh et al., 2014).

The bioactive compounds in *J. sabina* are of economic and health importance. The phenol catechin belongs to a family of polyphenols that have been reported to have anti-inflammatory (Monika et al., 2023), anti-tumour (Rawangkan et al., 2018; Shin et al., 2018), anti-diabetic (Silva et al., 2018; Spínola et al., 2019) and neuroprotective effects (Ortiz-López et al., 2016). Podophyllotoxins have received much attention for their good *in vitro* anticancer activity (Kumari et al, 2017). The *Juniperus* genus is a potential source of podophyllotoxins (Tavares and Seca, 2018). As many as 22 species of podophyllotoxins and their derivatives have been reported to be isolated and identified in *J. sabina* (Li et al., 2006). However, in the present manuscript, only a small number of podophyllotoxins were identified, including podophyllotoxin, deoxypodophyllotoxin, and podophyllotoxinone. The synthesis of podophyllotoxins is affected by factors such as growth environment, genetics, sampling time and location (Yang et al., 2016; Li et al., 2018). At the same time, this also shows the complexity of natural active products.

### 3.3 Validation of three enzyme inhibitory activities *in vitro*

This study analyzed the AChE, BChE, and α-glucosidase inhibitory activities of *J. sabina* extracts from various tissues (**Table 3**). The results revealed that the best inhibition results of AChE (47.37 μg/mL) and BChE (136.3 μg/mL) were shown in the leaves of adult *J. sabina*. However, extracts from *J. sabina* seedlings as well as branches and roots of adult *J. sabina* showed no significant inhibitory activity (>1000 μg/mL). In addition, all *J. sabina* extracts tested in this study were seen to have an inhibitory effect (62.59-412.43 μg/mL) on α-glucosidase (**Table 3**). Compared with cholinesterase, the stem (IC50 62.59 μg/mL) and branch (IC50 93.46 μg/mL) of biennial *J. sabina* showed the best α-glucosidase inhibitory activities, and they were only a few times higher than the positive control’s values (26.60 μg/mL). The stems and branches of young *J. sabina* appear to be more promising application tissues for α-glucosidase inhibitory activity.

**Table 3:** Enzyme inhibitory of extracts from different tissues of *J. sabina*.

Tree Ages	Tissues	AChE		BChE		α-Glucosidase	
		IC50 (μg/mL)	Inhibition % ± S.E.M. a	IC50 (μg/mL)	Inhibition % ± S.E.M. a	IC50 (μg/mL)	Inhibition % ± S.E.M. a
Biennial	Current year leaves	>1000	44.50 ± 1.29 a	750.34 ± 342.5	44.37 ± 1.87 ab	236.62 ± 1.8	80.45 ± 1.20 c
	Previous year leaves	>1000	36.10 ± 0.64 b	425.48 ± 136.42	47.50 ± 8.75 a	113.13 ± 3	91.73 ± 1.02 b
	Branch	>1000	31.37 ± 0.64 c	>1000	9.37 ± 5.62 d	93.46 ± 1.08	94.40 ± 0.71 a
	Current year stem	>1000	25.56 ± 2.15 d	>1000	30.00 ± 5.00 bc	62.59 ± 7.47	93.17 ± 0.29 ab
	Previous year stem	>1000	29.65 ± 0.21 c	>1000	31.25 ± 2.50 bc	159.05 ± 1.04	80.85 ± 0.13 c
	Root	>1000	30.51 ± 3.22 c	>1000	27.50 ± 15.00 c	132.05 ± 0.02	81.76 ± 0.38 c
Seven annual	Current year leaves	344.9 ± 9.46	50.63 ± 1.66 a	163.34 ± 104.35	56.96 ± 1.54 ab	412.43 ± 11.66	66.82 ± 0.82 e
	Previous year leaves	288.6 ± 10.22	50.08 ± 0.55 a	136.3 ± 59.73	62.07 ± 4.79 a	183.56 ± 3.35	82.39 ± 1.00 ab
	Branch	318.69 ± 70.25	50.08 ± 0.55 a	217.6 ± 77.77	53.93 ± 1.39 b	387.06 ± 6.15	70.34 ± 0.44 d
	Current year stem	>1000	38.15 ± 8.04 b	>1000	37.27 ± 2.47 d	110.78 ± 33.14	84.45 ± 3.65 a
	Previous year stem	>1000	41.97 ± 7.48 ab	>1000	45.47 ± 1.70 c	216.79 ± 9.76	79.57 ± 0.39 bc
	Root	>1000	32.56 ± 8.04 b	>1000	32.32 ± 3.40 d	184.82 ± 6.88	76.66 ± 0.18 c
Fifteen annual	Current year leaves	47.37 ± 6.79	73.98 ± 3.02 a	177.39 ± 6.92	60.12 ± 3.39 a	297.72 ± 17.01	78.61 ± 0.15 c
	Previous year leaves	160.76 ± 49.36	61.01 ± 4.53 b	327.19 ± 37.94	55.27 ± 4.03 a	247.57 ± 2.77	83.41 ± 1.20 ab
	Branch	416.69 ± 87.43	43.43 ± 2.11 c	210.44 ± 15.5	60.92 ± 2.90 a	405.44 ± 2.21	69.71 ± 1.27 d
	Current year stem	>1000	30.12 ± 2.11 e	>1000	35.92 ± 0.80 bc	104.89 ± 3.69	84.23 ± 0.68 a
	Previous year stem	>1000	34.96 ± 5.74 de	>1000	27.20 ± 10.17 c	172.07 ± 6.02	81.83 ± 0.65 b
	Root	>1000	38.59 ± 0.90 cd	>1000	39.15 ± 0.80 b	126.37 ± 0.62	84.49 ± 0.12 a
	Positive control	12.93 ± 2.47	70.96 ± 1.20	113.63 ± 8.8	73.12 ± 1.08	26.6 ± 0.69	95.67 ± 3.51

a Standard error mean (n=3); Sample concentration: 500 μg/mL; Positive control: Galantamine (for AChE 10 μg/mL; for BChE 100 μg/mL), Acarbose for α-Glucosidase 500 μg/mL, abcde represents significant differences in activity values when P< 0.05.

In the present study, it was found that the natural active constituents of *J. sabina*, along with other juniper plants, play an important role in the inhibitory properties of AChE, BChE, and α-glucosidase (Jung et al., 2015; Orhan et al., 2017; Tavares and Seca, 2018). The extracts of *J. sabina* in this study caused similar levels of AChE and BChE inhibition, comparable to extracts from other Juniperus leaves (Orhan et al., 2017; Tavares and Seca, 2018). And the inhibitory activity of *J. sabina* leaves on AChE and BChE may come from their lignans and phenolic compounds alone or in combination. Additionally, the results of α-glucosidase inhibitory activity in *J. sabina* extracts are higher than those of other *Juniperus* extracts (Kim et al., 2014; Orhan et al., 2017). At present, α-glucosidase inhibitors have become an important strategy for the treatment of diabetes (type II) (Cardullo et al., 2020). Flavonoids and biflavonoids in juniper seem to be the main factors to exert their hypoglycemic activity (Hee-Sung et al., 2023).

### 3.4 Anti-drug resistant bacteria activity *in vitro*

In this study, the microdilution method was used to determine the antibacterial activity of *J. sabina* extracts. The two clinically resistant strains were MRSA and VREC. **Table 4** showed that the antibacterial range of *J. sabina* extracts was 31.25-2000 μg/mL, and the antibacterial range of the positive control drug was 0.39-0.58 μg/mL. The order of antibacterial activity of the different tissues was root > previous year stem > current year stem > branch and leaves. The root extracts of fifteen annual *J. sabina* showed the best antibacterial activity against MRSA (MIC 31.25 μg/mL). However, the *J. sabina* extracts did not show significant inhibitory activity against VREC (MIC ≥500 μg/mL).

**Table 4:** Anti-drug resistant bacteria activity of extracts from different tissues of *J. sabina*.

Tree Ages	Tissues	MRSA	VREC
		MIC (μg/mL)	MIC (μg/mL)
**Biennial**	Current year leaves	1000 ± 0.00 a	1000 ± 0.00 a
	Previous year leaves	1000 ± 0.00 a	1000 ± 0.00 a
	Branch	1000 ± 0.00 a	750 ± 250.0 ab
	Current year stem	1000 ± 0.00 a	750 ± 250.0 ab
	Previous year stem	250.0 ± 0.00 b	1000 ± 0.00 a
	Root	62.50 ± 0.00 c	500.0 ± 0.00 bc
**Seven annual**	Current year leaves	1000 ± 0.00 a	1000 ± 0.00 a
	Previous year leaves	1000 ± 0.00 a	1000 ± 0.00 a
	Branch	1000 ± 0.00 a	1000 ± 0.00 a
	Current year stem	1000 ± 0.00 a	750.0 ± 250.0 b
	Previous year stem	187.5 ± 62.50 b	1000 ± 0.00 a
	Root	62.50 ± 0.00 c	2000 ± 0.00 a
**Fifteen annual**	Current year leaves	500.0 ± 0.00 a	1000 ± 0.00 a
	Previous year leaves	500.0 ± 0.00 a	1000 ± 0.00 a
	Branch	250.0 ± 0.00 b	1000 ± 0.00 a
	Current year stem	250.0 ± 0.00 b	750 ± 250.0 b
	Previous year stem	125.0 ± 0.00 c	750 ± 250.0 b
	Root	31.25 ± 0.00 d	750 ± 250.0 b
	Positive control	0.39 ± 0.00	0.58 ± 0.19

the positive control of MRSA was vancomycin hydrochloride; the positive control of VREC was colistin sulfate, abcde represents significant differences in activity values when P< 0.05.

The extracts from *Juniperus* branches, leaves, and berries showed broad-spectrum antibacterial properties, especially for *S. aureus* (Taviano et al., 2013; Dziedzinski et al., 2020). In addition, as key phenols and podophyllotoxins of the extracts, catechin, curcumin, and podophyllotoxin were previously shown to have inhibitory activity against *S. aureus* and *E. coli* in previous studies (Rocha et al., 2018; Muniz et al., 2021). However, the content of the above compounds in the roots of the best inhibiting tissues was low. Accordingly, the antibacterial activity of the different tissues of *J. sabina* cannot be simply attributed to the high content of phenolic and podophyllotoxins in the extracts. The antibacterial activity of *J. sabina* may be influenced by other podophyllotoxin, phenol and undetected components, or by synergistic effects between them.

### 3.5 Chemometrics analysis of *J. sabina* samples

In order to investigate the main factors influencing the biological activity of the extracts of *J. sabina*, the values of the inhibition rates of the three enzymes and the MIC values of the antibacterial activity were selected for analysis in relation to the content and composition of the extract compounds. As the content of podophyllotoxin and catechin showed a significant linear correlation (VIF > 10) with the bioactivity of *J. sabina*, nine compounds other than them were selected for Redundancy analysis (RDA) with bioactivity in this study. The RDA1 and RDA2 explained 90.80% and 6.61% of the variance, respectively. Obtuse angles indicate a negative correlation, and acute angles indicate a positive correlation. The results of the RDA analysis (**Figure 4**) showed that the living activity of the extract of *J. sabina* showed a close correlation with epicatechin and matairesinol. Studies have shown that epicatechin and matairesinol have positive effects on antibacterial, anti-acetylcholinesterase, anti-α-amylase, anti-butyrylcholinesterase and anti-α-glucosidase activities (Kim et al., 2016; Bursal et al., 2019). In addition, the clustering results of samples showed that the biological activity of *J. sabina* samples was closely related to their age, and adult *J. sabina* seemed to have better application potential than younger *J. sabina*, which might be related to the rich accumulation and composition of active compounds in adult *J. sabina*.

## 4 Conclusions

The present study reports for the first time the phenolic and lignan content, compositional characteristics, and biological profile of the methanol extract of *J. sabina*. The leaves and roots of fifteen annual *J. sabina* contain the highest levels of total lignans and total phenols. High levels of TPC (87.16 mg GAE/g DW) and TLC (491.24 mg PPT/g DW) were detected in fifteen annual *J. sabina*. Eleven phenols and lignans were identified by HPLC/HPLC-MS, which were mainly distributed in the leaves of adult *J. sabina*. Studies on the biological activities found that the extracts of seven annual *J. sabina* roots and fifteen annual *J. sabina* current year leaves had the potential to be anti-MRSA and neuroprotective agents, respectively. In addition, the extract of biennial *J. sabina* branches possessed excellent anti-diabetic activity, and its exact mechanism could be further investigated. The results of the RDA analysis showed that the biological activity of the extract of *J. sabina* showed a close correlation with epicatechin and matairesinol. The results of this study support the potential application value of *J. sabina* in neuroprotection, diabetes, and anti-drug resistance bacteria. And it will help facilitate the application of *J. sabina* resources in the functional food, pharmaceutical, and nutraceutical industries.

## Data availability statement

The original contributions presented in the study are included in the article/**Supplementary Material.** Further inquiries can be directed to the corresponding author.

## Author contributions

SX: Methodology, Software, Formal analysis, Writing - Original Draft. QC: Software, Formal analysis, Resources, Writing - Review & Editing. NL: Resources, Data Curation. JY: Formal analysis, Investigation, Supervision. DL: Conceptualization, Methodology, Supervision. All authors contributed to the article and approved the submitted version.
