# Supplementary material for: Effects of age and tissue of Juniperus sabina L. on its phytochemical characteristics, anti-cholinesterase, antidiabetes, and anti-drug resistant bacteria activities
Source: Front Plant Sci. 2023 Sep 4;14:1174922. doi: 10.3389/fpls.2023.1174922 (PMC10507269; doi:10.3389/fpls.2023.1174922)
Supplement: Supplementary file 1 [file DataSheet_1.docx]

**Supplementary material**

**Tables**

**SM-Table 1** The yields (%, w/w) of *J. sabina* extracts

| **Age** | **Tissue** | **Yield (%)** |
| --- | --- | --- |
| **Biennial** | Current year leaves (BCL) | 28.55 |
|  | Previous year leaves (BPL) | 27.2 |
|  | Branch (BB) | 21.375 |
|  | Current year stem (BCS) | 10.425 |
|  | Previous year stem (BPS) | 8.95 |
|  | Root (BR) | 13.125 |
| **Seven annual** | Current year leaves (SCL) | 32.1 |
|  | Previous year leaves (SPL) | 26.925 |
|  | Branch (SB) | 19.15 |
|  | Current year stem (SCS) | 11.125 |
|  | Previous year stem (SPS) | 10.95 |
|  | Root (SR) | 10.325 |
| **Fifteen** **annual** | Current year leaves (FCL) | 34.55 |
|  | Previous year leaves (FPL) | 28.15 |
|  | Branch (FB) | 20.5 |
|  | Current year stem (FCS) | 12.375 |
|  | Previous year stem (FPS) | 8.975 |
|  | Root (FR) | 10.675 |

**SM-Table 2** MRM transitions and MS conditions for the identification of compounds

| **Compounds** | **CXP** | **EP** | **IS** | **Transitions** |
| --- | --- | --- | --- | --- |
| **Catechin** | 10 | 10 | 5500 | 1806 |
| **Epicatechin** | 10 | 10 | 5500 | 1806 |
| **Luteolin** | 10 | 10 | 5500 | 1806 |
| **Apigenin** | 10 | 10 | 5500 | 1806 |
| **Amentoflavone** | 10 | 10 | 5500 | 1806 |
| **Curcumin** | 10 | 10 | 5500 | 1806 |
| **Podophyllotoxinone** | 10 | 10 | 5500 | 1806 |
| **Podophyllotoxin** | 10 | 10 | 5500 | 1806 |
| **Matairesinol** | 12 | 10 | 4500 | 392 |
| **Deoxypodophyllotoxin** | 10 | 10 | 5500 | 1806 |
| ***α*-Peltatin** | 10 | 10 | 5500 | 1806 |

**SM-Table 3** Details of the standard curve

| **No.** | **Analytes** | **Regression equation** | ***R*^2^** | **Liner range(μg/mL)** | **LOD (μg/mL)** | **LOQ (μg/mL)** |
| --- | --- | --- | --- | --- | --- | --- |
| 1 | Cianidanol | y = 2373.4x - 0.1403 | 0.9993 | 15.63-500.00 | 4.43 | 15.63 |
| 2 | Epicatechin | y = 3850.1x - 44.75 | 0.9954 | 15.63-500.00 | 4.36 | 15.63 |
| 3 | Matairesinol | y = 1210.3x + 0.342 | 0.9994 | 31.25-1000.00 | 9.47 | 31.25 |
| 4 | α-Peltatin | y = 1481.3x + 4.6057 | 0.9987 | 5.21-500.00 | 4.47 | 15.63 |
| 5 | Luteolin | y = 39426x - 19.354 | 0.9999 | 1.95-62.50 | 0.67 | 1.95 |
| 6 | Podophyllotoxin | y = 9736x + 9.1552 | 0.9946 | 15.63-500.00 | 5.21 | 15.63 |
| 7 | Podophyllotoxinone | y = 38463x - 6.5851 | 0.9996 | 1.95-62.50 | 0.57 | 1.95 |
| 8 | Apigenin | y = 54267x + 17.773 | 0.9999 | 1.95-62.50 | 0.66 | 1.95 |
| 9 | Deoxypodophyllotoxin | y = 22774x + 5.4356 | 0.9995 | 3.96-125.00 | 1.24 | 3.96 |
| 10 | Amentoflavone | y = 90596x - 42.296 | 0.9999 | 3.96-125.00 | 1.07 | 3.96 |
| 11 | Curcumin | y = 13571x - 35.339 | 0.9996 | 15.63-500.00 | 5.12 | 15.63 |

**Figures**


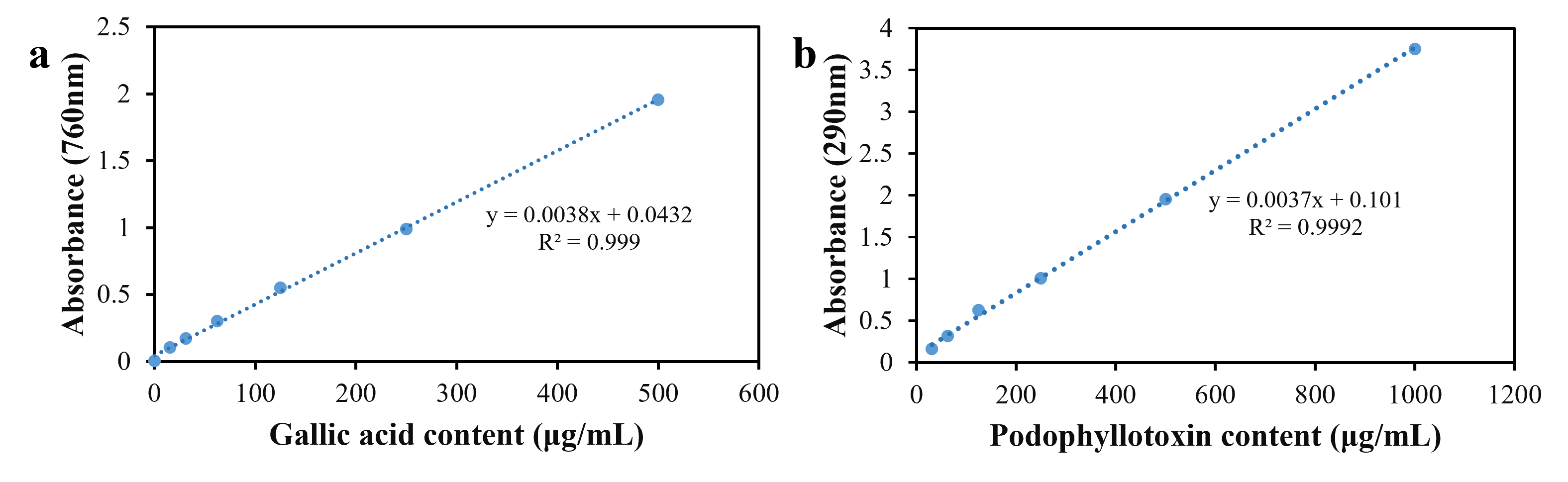


**SM-Figure. 1** Standard curves for gallic acid and podophyllotoxin


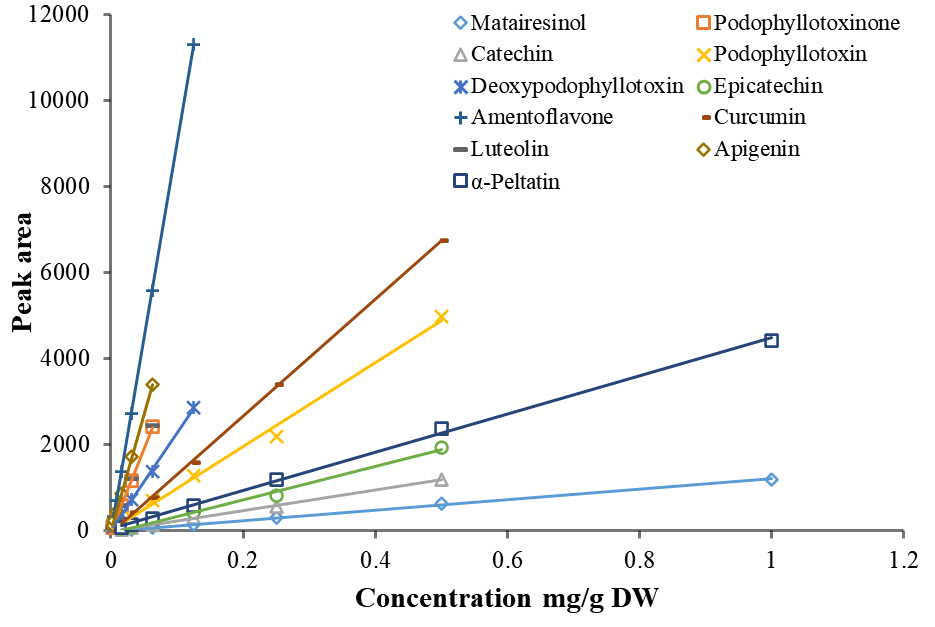
**SM-Figure. 2** Standard curves of 11 compound standards


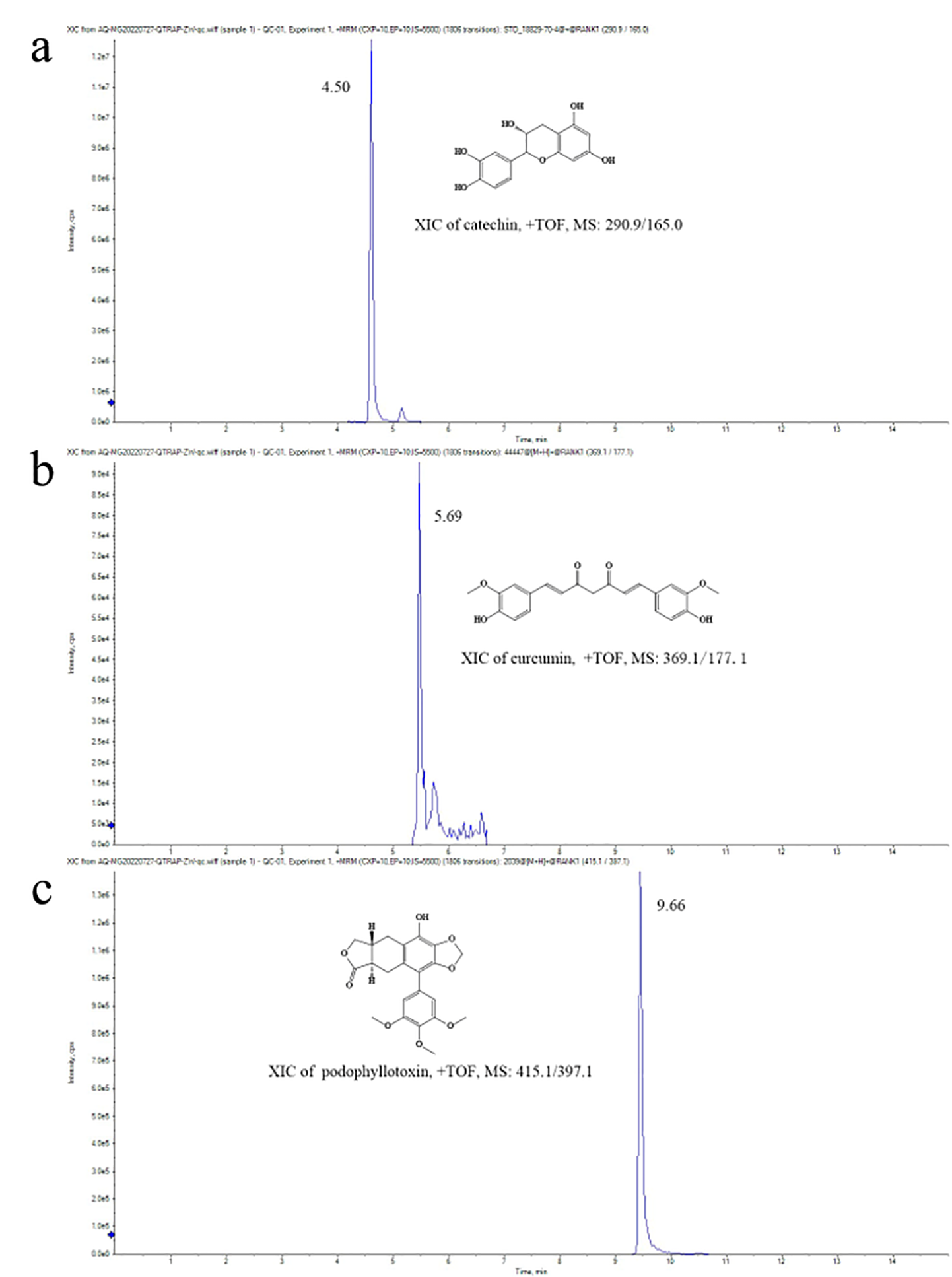


**SM-Figure. 3** Extracted ion chromatogram of LC-QTOF-MS


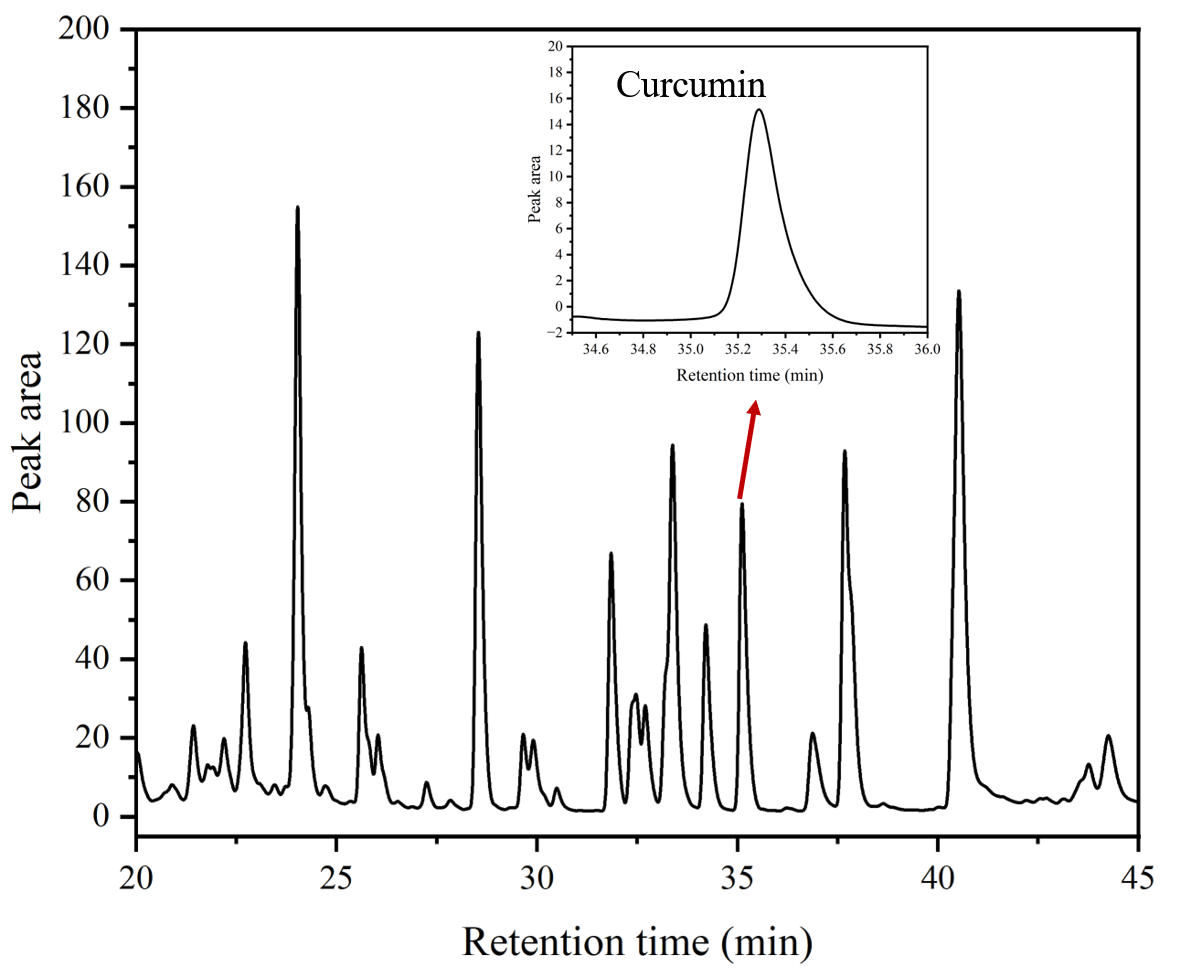


**SM-Figure. 4** Liquid chromatogram of *J. sabina* leaves extract and curcumin standard
